# Report of the Committee of the American Gynecological Society

**Published:** 1910-07

**Authors:** 


### TEACHING OF OBSTETRICS

### Report of the Committee of the American Gynecological Society

### Present Status of Obstetrical Education in Europe and America, and Recommendations for the Improvement of Obstetrical Teaching in America

[Concluded from the June number]

COLUMBIA UNIVERSITY, COLLEGE OF PHYSICIANS AND SURGEONS, MEDICAL DEPARTMENT.

*Course in Obstetrics.*

Second Year. Recitations and Demonstrations (once a week for 30 weeks), 30 hours.

Third Year. (First Half). Didactic Lectures (twice a week for one-half year), 30 hours; Clinical Lectures (once a week for one-half year), 15 hours.

Fourth Year. Practical Instruction in Hospital and Tenements.

(a) Three weeks service in hospital; two weeks being spent on day duty and one week on night duty. During this term of service each student receives daily bedside instruction and makes antepartum examinations, both abdominal and vaginal, on from 50 to 60 pregnant women. Moreover the students on duty receive a daily clinical lecture and manikin instruction from an instructor in obstetrics who is the resident obstetrician.

(b) Two weeks service in the tenements; one week being spent on day duty and one week on night duty.

Each student during his five weeks of practical service delivers personally on an average seven or eight cases and sees from forty to fifty deliveries.

COLUMBIA UNIVERSITY.

*Course in Gynecology.*

Third Year. First Half. Recitations (once a week for 15 weeks), 15 hours. Second Half. Didactic lectures (twice a week for 15 weeks), 30 hours. Clinical lectures (once a week for 15 weeks), 15 hours.

Fourth Year. Practical instruction in small sections in dispensary and hospital (26 hours for each student), 26 hours.

(Signed) E. B. CRAGIN.

CORNELL UNIVERSITY MEDICAL COLLEGE, NEW YORK CITY.

*Plan of Instruction in Obstetrics, January, 1910.*

Second Year. Recitations (32 hours), 32 hours.

Third Year. Section and Manikin Work (16 hours); Clinics (16 hours); Illustrative Lectures (32 hours); Recitations (32 hours); 96 hours.

Fourth Year. Clinics (16), total, 144 hours.

In addition students are required to reside for at least two weeks in the Manhattan Maternity or other hospital and personally confine at least six women.

J. CLIFTON EDGAR.

HARVARD MEDICAL SCHOOL, MEDICAL DEPARTMENT OF HARVARD UNIVERSITY.

*Department of Obstetrics and Gynecology.*

A. Course in Obstetrics.

Third Year. Lectures on the theory and practice of obstetrics (twice a week), 64 hours; recitations (once a week), 32 hours; conferences (once a week), 32 hours; clinical instruction. Each student spends two weeks in hospital residence, devoting his whole time, day and night, to his obstetric opportunities. He sees operation and normal deliveries and under supervision and instruction he personally attends from six to ten out-patient cases. After his two weeks of residence he is required to devote a part of his time for a week or more to completing the visits on his patients and writing reports of his cases.

Fourth Year.—(In the Harvard Medical School the work of the fourth year is elective; but all students intending to practise medicine elect obstetrics.)

The class work in sections of from six to ten, and each student in obstetrics devotes his entire time for a month. For two weeks he is in hospital residence, and attends from six to ten out-patients, under supervision and instruction. After his period of residence, he completes the visits of convalescence and reports on his cases. There is a clinical lecture and ward visit every forenoon (except Sunday), at which the student has opportunity for antepartum examinations (inspection, palpation, auscultation, pelvimetry, and estimates of size of fetus, for witnessing normal and operative deliveries, for studying puerperal convalescence and the care of young infants. Each student has also a course of instruction, with manikin and fetal cadaver, in which the various obstetric operations are demonstrated and repeated by the student. Each student also writes a thesis on an approved subject of his choice.

(Many of the Harvard students make use of the opportunities afforded by the summer courses of the Harvard Medical School, and thus increase their clinical training. In addition to the many cases witnessed, the graduates of 1909 attended personally an average of twenty-three cases.)

B. Course in Gynecology.

Third Year. (Second Half). Lectures or recitations (twice a week), 32 hours; Clinical exercises in small sections. Each student attends six clinics, lasting from one and one-half to two hours. In these clinics the student is instructed in physical examination, diagnosis, and the treatment of ambulatory cases.

Fourth Year (elective, taken by a large part of the class). Instruction is given in sections of from six to ten students, and each student devotes his entire time during the forenoons of two months. The work is clinical, and is given in the wards and out-patient department of the Boston City Hospital. Opportunity is afforded for practice in history taking, examination, diagnosis, and minor treatment in the out-patient department. In the House Service the student hears clinical lectures daily, has opportunity for physical examinations, and witnesses operations with demonstration; he follows the convalescence of cases, and each in turn assists in the work of the resident staff. Each student also has abundant opportunity for the study, under supervision, of pathological specimens removed in his presence by operation, and each student writes a thesis on an approved subject of his choice.

(Signed) C. M. Green.

JEFFERSON MEDICAL COLLEGE, PHILADELPHIA.

*Course in Obstetrics.*

The anatomy and physiology of reproduction fully taught by the departments of anatomy and physiology in the first two years. Embryology and histology are included in this teaching.

Third Year. Three didactic lectures and recitations (weekly), 90 hours; demonstration with the manikin and diagnosis, obstetric manipulations and vaginal deliveries, 18 hours. At least one case of spontaneous parturition in hospital, fully demonstrated by an instructor.

Fourth Year. Lectures to the entire class (one weekly), 30 hours; hospital ward classes with the examination of pregnant patients, the study of complications of pregnancy the puerperal period, normal infancy and complications, 16 hours; clinical conferences in hospital with study of cases, 24 hours; demonstrations of hospital cases by instructors to small groups of students, 16 hours; from two to six cases delivered in tenements and under supervision and instruction.

Written reports of these cases with quizzes upon the reports by a demonstrator.

Record of all work done during the senior year, which record with final examination constitutes final grade for securing a degree.

E. P. DAVIS.

JOHNS HOPKINS UNIVERSITY, BALTIMORE.

*Courses in Obstetrics.*

Third Year: obligatory course. Recitations and demonstrations twice weekly for 33 weeks, 66 hours; manikin work, once a week for 33 weeks, 33 hours. Ward rounds and clinics in groups, once a week for 16 weeks, 16 hours; examination of pregnant patients in groups once a week for 16 weeks, 16 hours, total 132 hours; obligatory attendance of at least 5 cases of labor under supervision in the ward; optional work and courses in obstetrical histology and pathology, two hours a week for 11 weeks, 22 hours.

Fourth Year: elective work. Repeated every 11 weeks to not more than 10 students each time. Each course occupies 99 hours, not including obligatory attendance on at least 10 cases of labor in the out-patient department and attendance at as many operations in the ward as feasible. The course consists of: ward rounds, 11 hours; conferences, 11 hours; discharge examination of puerperal women, 11 hours. A practical course in pelvimetry, 11 hours; a laboratory course in infant feeding, 11 hours; nursery rounds, 11 hours; A practical and laboratory course on the toxemias of pregnancy, 22 hours; a course in comparative placentation, 11 hours.

I might add that many of the students in these groups see from 25 to 40 out-door deliveries. In each case they are accompanied by an assistant and a trained nurse, and I find that such training is even more valuable than the ward deliveries. They also make visits for the first five, the seventh and tenth days of the puerperium in normal cases, and as many visits as may be necessary in abnormal cases.

These visits are checked in two ways, first, by having the student leave a daily written report in the letter box of the resident obstetrician, and secondly, by having the nurse, who makes daily visits for ten days render a similar report.

J. W. Williams.

UNIVERSITY OF CHICAGO.

The subjects of obstetrics and gynecology are taught in the junior and senior years in laboratory, recitation, and conference courses, in dispensary and hospital clinics, and in the conduct of labor in the homes of patients. Students are obliged to commence their studies by taking the laboratory and recitation courses. Final examinations in both courses are compulsory.

*Obstetrics.*

1. Conference course on normal pregnancy, labor, and the puerperium. A lecture and recitation course. Each section limited to forty students.

2. Clinical conference on normal pregnancy, labor and the puerperium. Prerequisite: course 1. Limited to forty students.

3. Clinical conference on the pathology of pregnancy, labor and the puerperium. Prerequisite: courses 1 and 2. Limited to twenty-five students.

*Senior Year:*

4. Practical obstetrics. Prerequisite: courses 1, 2 and 3. Limited to fifteen students.

Clinical Obstetrics.—In the maternity department of the Presbyterian Hospital, Charity Hospital, Chicago Lying-in Dispensary, Chicago Maternity, and Central Free Dispensary. Prerequisite: courses 1 and 2. Throughout the year. Attendance upon cases of confinement in various hospitals, and at the homes of patients is required of each student before graduation. Each student will be summoned to cases at the time of delivery, and will attend the patients during and after delivery, under supervision. Clinical records must be kept by students and certificates obtained for attendance on five cases.

*Gynecology.*

*Junior Year:*

6. Laboratory and Recitation Courses: Limited to twenty-five students.

*Junior and Senior Year:*

7. Clinical Conference.—Prerequisite: course 6. Limited to forty students.

8. Dispensary Clinics.—Conferences in practical gynecology, limited to four in each section. Prerequisite: course 6. Twenty-four hours. Four M. Each term throughout the year.

*Senior Year:*

9. College Clinics.—In gynecology and obstetrics. Prerequisite: course 6. Forty-eight hours. Four Mj. Each quarter throughout the year.

10. Special Laboratory Work.—For a limited number of students selected by the department staff.

Our teaching methods have been gradually changing in the last ten years. Systematic lectures have been entirely or almost entirely abolished and we have endeavored to instruct our students in small classes. Twenty-two majors of work are required in the junior and senior years, three being necessary in obstetrics and gynecology (at least two majors in obstetrics are required). Most students voluntarily take more than the requisite three majors.

The faculty feels strongly that there should be an extra fifth year in which more clinical instruction could be given. However, as all our graduates are able to obtain interneships, we feel that we are better off than most medical schools.

The enclosed statement of departmental work gives a detailed account of our method of instruction.

We feel that the number of obstetric cases which should be attended by students is too small. It should be at least twelve. We intend to increase this requirement as our clinical facilities improve.

J. C. WEBSTER.

### UNIVERSITY OF PENNSYLVANIA, MEDICAL DEPARTMENT.

### *Course in Obstetrics.*

Third Year. Clinical lectures twice a week, 60 hours; demonstrations of abdominal palpation, pelvimetry, etc., to individual students, each, 1 hour; attendance on a patient in the hospital under supervision and visits daily for two weeks afterward, average, 24 hours; recitations—voluntary (quiz).

Fourth Year. One clinical lecture a week for half the year, 18 hours; two weeks of ward class instruction for two hours a day, 24 hours; six demonstrations on the manikin to sections, 6 hours; one week's residence in the South-Eastern Dispensary for out-patient work. Number of labors attended by each student—average 7; recitations—voluntary (quiz).

Scope of instruction.—The physiology and pathology of the childbearing process including all the complications and pathological consequences at all periods and their treatment, medical and surgical.

B. C. Hirst.

*Recommendations.*

We recommend that the teaching of obstetrics should occupy at least two years of the medical course and that those expecting to practise obstetrics, should be urged to avail themselves of elective opportunities.

That the number of labor cases personally attended by each undergraduate student should be at least six; under supervision and instruction.

*Character of Instruction.*

We recommend all the known methods of teaching this branch of medicine, namely: didactic lectures, clinical lectures, clinical conferences, ward classes and touch courses, hospital and out-patient instruction, manikin practice in operative obstetrics and recitation.

Of the first three methods, we recommend specially, clinical lectures and conferences.

We recommend that ample facilities should be afforded students to make antepartum examinations, including inspection, abdominal palpation, pelvimetry, fetometry, vaginal examinations, etc.

We recommend that a two weeks' hospital residence should be required before the out-patient practice.

*Scope of Instruction.*

It is recommended that as obstetrics at present includes pregnacy and parturition, their complications and consequences and the complete recovery of the women after labor; that obstetric instruction should include the medical and surgical treatment of these conditions.

The tendency of obstetrics to become more surgical in practice and to require a surgical training, is evidenced by the fact that in the medical schools of Europe and in more than one-third of the first fifteen medical colleges of this country, the chairs of obstetrics and gynecology are combined under one head —namely, Columbia, Cornell, Jefferson, Medico-Chirurgical, Tulane, Yale, Long Island, Harvard, Johns Hopkins, Rush, Bellevue, Western Reserve, Michigan, University of Pennsylvania, California.

Of these fifteen medical schools, six have combined chairs.

Signed.

E. B. Cragin,

